# Classification of distal radius fractures in children: good inter- and intraobserver reliability, which improves with clinical experience

**DOI:** 10.1186/1471-2474-13-6

**Published:** 2012-01-23

**Authors:** Per-Henrik Randsborg, Einar A Sivertsen

**Affiliations:** 1 The Department of orthopaedic surgery, Akershus University Hospital, Lørenskog, Norway; 2 Faculty Division, University of Oslo, Norway

## Abstract

**Background:**

We wanted to test the reliability of a commonly used classification of distal radius fractures in children.

**Methods:**

105 consecutive fractures of the distal radius in children were rated on two occasions three months apart by 3 groups of doctors; 4 junior registrars, 4 senior registrars and 4 orthopedic consultants. The fractures were classified as buckle, greenstick, complete or physeal. Kappa statistics were used to analyze inter- and intraobserver reliability.

**Results:**

The kappa value for interobserver agreement at the first reading was 0.59 for the junior registrars, 0.63 for the senior registrars and 0.66 for the consultants. The mean kappa value for intraobserver reliability was 0.79 for the senior registrars, 0.74 for the consultants and 0.66 for the junior registrars.

**Conclusions:**

We conclude that the classification tested in this study is reliable and reproducible when applied by raters experienced in fracture management. The reliability varies according to the experience of the raters. Experienced raters can verify the classification, and avoid unnecessary follow-up appointments.

## Background

Distal radius fractures is the most common fracture in childhood [1]. Most of these fractures are treated conservatively in a plaster and complications are rare. Although these fractures generally are benign, they are monitored differently according to the stability of the fracture and whether the growth plate is injured or not. A clinically relevant classification must take these factors into account. Otherwise the classification will not be helpful in deciding the correct treatment, follow-up strategy and prognosis. Any fracture classification should also have a substantial degree of both inter- and intraobserver reliability. If not, treatment algorithms will be arbitrary, since the fractures are placed in different categories by different doctors. If an unstable fracture is classified in a benign category with little or no follow-up, it can lead to complications, i.e. malunion of the fracture. Placing stable fractures, such as buckle fractures, in categories for unstable fractures will cause more follow-up than necessary. This is costly both for patients and society. On the other hand, a fracture classification with high reliability will provide effective, predictable and safe treatment algorithms, and it will be possible to draw general conclusions from research based on that system.

Very few commonly used fracture classifications have demonstrated acceptable inter- and intraobserver agreement [2-5]. This is certainly the case for distal radius fractures in adults [6-11] (Table 1). However, pediatric fractures are different from adult fractures. The pediatric bone has a thick periosteal sleeve, is softer and more pliable than adult bone and the growing bone exhibits the unique feature of growth with considerable remodeling potential [12]. These differences make adult fracture classifications unsuitable for pediatric fractures.

**Table 1:** Reported reliability of different fracture classifications for distal radius fractures in adults

Reference	Classification	Interobserever reliability	Intraobserver reliability
Kreder et al 1996	AO type	0.68	0.86
	AO group	0.48	
	AO subgroup	0.33	0.42
Ploegmakers et al 2007	AO/ASIF	0.10*	0.52
	Frykman	0.10*	0.26
	Fernandez	0.16*	0.42
	Older	0.15*	0.27
Jin et al 2007	AO type	0.45	0.49
	AO group	0.25	0.36
	Frykman	0.36	0.54
	Cooney group	0.59	0.72
	Cooney subgroup	0.36	0.42
Andersen et al 1996	Frykman	0.36	0.48
	Melone	0.34	0.48
	Mayo	0.43	0.44
	AO type	0.64	0.66
	AO Group	0.30	0.37
	AO subgroup	0.25	0.31
Flinkkilä et al 1998	AO/ASIF modified	0.23	
Belloti 2008	AO/ASIF	0.27	0.49
	Frykman	0.24	0.55
	Fernandez	0.34	0.59
	Universal (Cooney)	0.40	0.61

Kappa coefficients unless where indicated

* Spearman rank correlation coefficient

**Figure 1:**
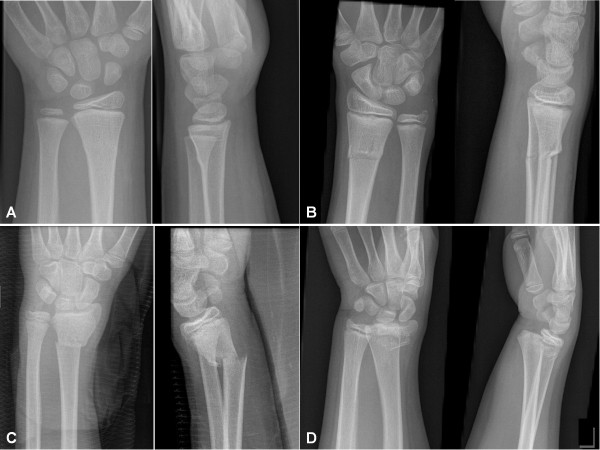
**Examples of fractures from each category**. A: Buckle fracture, rated as buckle on all readings. B: Greenstick fracture, rated as greenstick on 20 of 24 readings. C: Complete fracture, rated as complete on all readings. D: Physeal fracture, as rated by all raters on both readings

Fractures of the distal radius in children are commonly grouped into four categories [13-21] (Figure 1): Buckle (torus) fractures are characterized by a compression failure of bone without disruption of the cortex on the tension side of the bone [14]. The greenstick fractures differ from the buckle fracture as the cortex is disrupted on the tension side, but intact on the compression side of the fracture [19]. Complete fractures (adult type) have disruption of both cortices in one plane. Physeal injuries occur frequently during the preadolescent growth spurt, when there is a transient cortical porosity caused by the increase in calcium requirement and bone turn-over [22]. Fractures involving the growth plate are often subdivided according to the classification of Salter and Harris [23]. The follow-up algorithm of these different categories varies, thus the classification will provide guidelines for management and prognosis. Buckle fractures are stable, and don't need follow up, while the lateral angulation of greenstick fractures often change during the immobilization period [18,24,25]. Complete fractures are highly unstable, and will often need fixation with Kirschner pins [15,16]. Fractures involving the physis might lead to growth disturbances, although this is rare. The risk of growth disturbances increase, however, if the fracture is reduced more than 3 days after the fracture, or if repeat attempts of reduction is attempted [13].

The aim of this study is to evaluate the interobserver and intraobserver reliability of this commonly used classification of distal radius fractures in children. Also, we wanted to investigate to what degree experience influence the reliability of the classification. To the best of our knowledge this has not previously been done, although this classification has been used in several publications.

## Methods

We designed this study to comply with the guidelines for reliability studies of fracture classification systems outlined by Audigé, Bhandari and Kellam [26]. We included the first 105 consecutive distal radius fractures in children below the age of 16 years treated at our institution in 2007. Where indicated, information on follow ups was retrieved from the electronic journal. The radiographs were identified via our computerized files and checked by the authors. Fractures not involving the metaphysis according to the AO Pediatric Comprehensive Classification of long bone fractures were considered to be diaphyseal fractures and were excluded from the study [27]. No radiograph was excluded due to poor quality, to avoid selection bias. Standard anterior-posterior and lateral radiographs of the distal radius were reviewed independently by 12 different observers: four junior orthopedic residents with a mean experience of fracture management of 14 months (6-28), four senior orthopedic registrars with an average experience in fracture management of 41 months (30-49) and four experienced orthopedic surgeons. In our institution, the pediatric distal radius fractures are generally managed by the junior registrars, who are supervised by the senior registrars. The orthopedic consultants are only occasionally involved in the management of these fractures.

Each fracture was classified to one of four possible categories; buckle (or torus), greenstick, complete or physeal fracture. The physeal fractures were not subclassified further. Before rating the radiographs, the observers were given schematics of the different fracture types and a written introduction to the difference between the categories. Further instructions to enhance results were not given. The radiographs were reviewed by the observers at two occasions 3 months apart. The raters were blinded to clinical information regarding the patients. The observers were not given any feed-back between the observations, and the order of the fractures was randomly changed before the second rating.

### Statistics

The statistical analyses were performed using the free software R version 2.9.2 [28] and the associated package irr [29]. Kappa statistics were used to analyze interobserver and intraobserver variation. The Kappa value is a coefficient of agreement between observers, correcting for the proportion of agreement that could have occurred by chance. Fleiss introduced a category-specific kappa score and a kappa score that can be employed when there are more than two observers, as is the case in our study [30]. For intraobserver variation we used Cohens kappa [31]. A kappa score of 1 indicates perfect agreement and a score of zero indicates that the variation in agreement can be explained purely by chance. Several authors have provided guidelines to the interpretation of kappa scores [30,32,33] (Table 2).

**Table 2:** Interpretation of kappa values according to different authors

Kappa value	Fleiss	Svanholm	Landis and Koch
0.95 - 1.00			
			
0.90 - 0.95			ALMOST
			
0.85 - 0.90	EXCELLENT	EXCELLENT	PERFECT
			
0.80 -0.85			
			
0.75 - 0.80			
	
0.70 - 0.75			SUBSTANTIAL
			
0.65 - 0.70			
			
0.60 - 0.65	FAIR	GOOD	
			
0.55 - 0.60	TO		
			
0.50 - 0.55	GOOD		MODERATE
			
0.45 - 0.50			
			
0.40 - 0.45			
		
0.35 - 0.40			
			
0.30 - 0.35		POOR	FAIR
			
0.25 - 0.30			
			
0.20 - 0.25			
			
0.15 - 0.20	POOR		
			
0.10 - 0.15			SLIGHT
			
0.05 - 0.10			
			
0.00 - 0.05			

## Results

### Interobserver reliability

**Figure 2:**
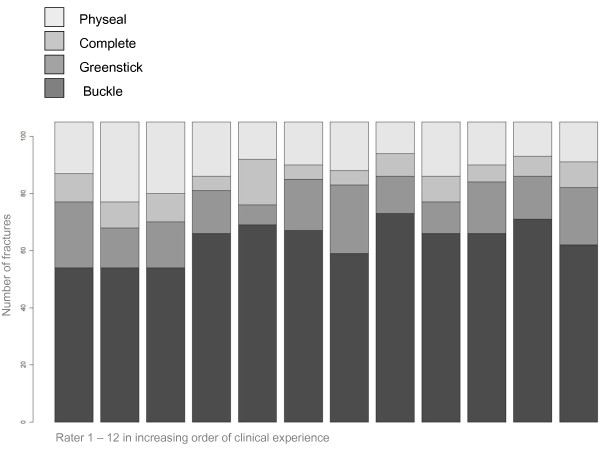
**Classification of 105 consecuitive pediatric distal radius fractures into four categories by 12 raters in increasing order of clinical experience**.

The highest interobserver agreement at the first reading was achieved by the consultants, with a percentage of agreement of 67.6%, and a kappa value of 0.66 (Table 3). The overall interobserver agreement among the 8 most experienced observers was 58.1% of the radiographs at the first reading, representing a kappa value of 0.66 (95% C.I: 0.64 - 0.68). This was better than the junior registrars (p < 0.01) with 56.2% agreement at the first reading, producing a kappa value of 0.59 (95% C.I: 0.54 - 0.64). The difference in rating between the doctors is demonstrated in Figure 2.

**Table 3:** Reliability of fracture classification of 105 consecutive pediatric distal radius fractures rated by 12 doctors with variable level of experience in fracture management.

	Complete agreement at first reading	Two categories at first reading	Three categories at first reading	Four categories at first reading	Inter-observer values at first reading	Inter-observer values at second reading	Intra-observer agreement (Mean kappa)
All 12 raters	41.9	40.0	15.2	2.9	0.61	0.59	0.73
8 senior raters	58.1	30.5	11.4	-	0.66	0.70	0.77
4 junior registrars	56.2	38.1	5.7	-	0.59	0.50	0.66
4 senior registrars	65.7	27.6	6.7	-	0.63	0.72	0.79
4 consul-tants	67.6	27.6	4.8	-	0.66	0.67	0.74

Kappa values and percentage of cases placed in one, two, three or all four categories

The greenstick and complete fractures were most difficult to agree on, while buckle fractures had the highest category-specific kappa value (Table 4). No greenstick fracture was rated as such by all raters on both readings.

**Table 4:** Category-specific kappa values at first reading

Fracture type	Junior registrars	Junior registrars	Senior registrars	Consultants
Buckle	0.63	0.75	0.72	
Greenstick	0.47	0.47	0.52	0.52
Complete	0.57	0.40	0.66	
Physeal	0.64	0.64	0.66	0.70

### Intraobserver reliability

**Figure 3:**
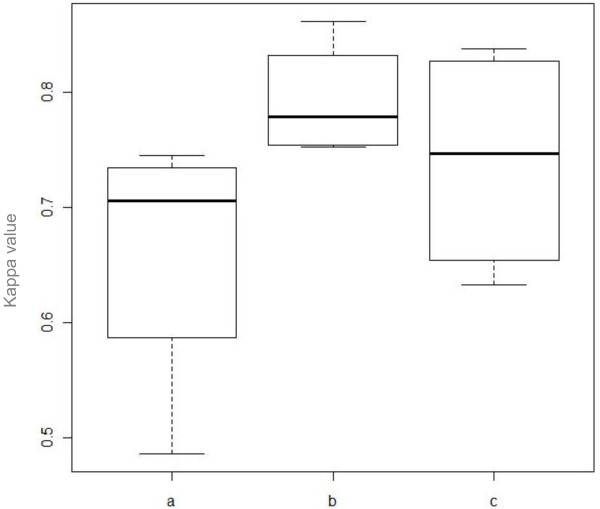
**Box-plot demonstrating the difference in intraobserver agreement between a) junior registrars, b) senior registrars and c) consultants**.

The highest mean intraobserver kappa value was achieved by the senior registrars; with a mean kappa value of 0.79 (Table 5). The junior registrars had the lowest score with a mean kappa value of 0.66. The mean kappa value for the consultants was 0.74. Figure 3 illustrates the difference in intraobserver agreement between the three groups of raters.

**Table 5:** Intraobserver agreement for 12 raters with different experience in fracture management

Rater	Number of months in practice	Cohens kappa value	Percentage of agreement
1 Junior registrar	< 6	0.49	62.9
2 Junior registrar	7	0.69	81.0
3 Junior registrar	18	0.75	83.8
4 Junior registrar	28	0.72	83.8
5 Senior registrar	30	0.75	86.7
6 Senior registrar	38	0.76	86.7
7 Senior registrar	46	0.80	88.6
8 Senior registrar	49	0.86	93.3
9 Consultant	89	0.68	81.0
10 Consultant	105	0.84	91.4
11 Consultant	110	0.82	90.5
12 Consultant	> 200	0.63	78.1

### Distribution of fractures

We defined a buckle fracture as a fracture categorized as buckle by at least 6 of the 8 most experienced doctors on both readings. We identified 65 such fractures. 53 of these stable fractures were given a clinical and/or radiological follow-up appointment. Only 10 patients were told to remove the splint themselves after 3 weeks with no appointment scheduled (table 6). All healed uneventfully. The distribution of the fracture categories based on consensus among the raters as well as the distribution of age and sex is presented in Table 7.

**Table 6:** Follow up of 65 buckle fractures

Type of follow up	Number of patients	Number of clinical follow-ups	Number of radiological examinations
No follow up scheduled	10	0	0
After 1 week only	3	3	2
After 1 week and at plaster removal	20	40	30
At plaster removal only	29	29	2
Unknown	3		

**Total**	**65**	**72**	**34**

The fractures were defined as a buckle if it was rated as such by at least 6 of the 8 most experienced doctors on both readings

**Table 7:** Classification of 105 consecutive distal radius fractures by consensus among 12 raters, including age and gender distribution.

Fracture type	Number of patients	Number of boys	Mean age in years (range)
Buckle	65	45 (69%)	11.0 (1.6 - 15.8)
Greenstick	18	12 (67%)	12.0 (7.3 - 15.4)
Complete	6	5 (83%)	13.7 (12.6 - 14.8)
Physeal	16	10 (63%)	12.2 (6.0 - 15.8)

**Total**	**105**	**72 (69%)**	**11.4 (1.6 - 15.8)**

## Discussion

The overall interobserver reliability of this fracture classification is better than most other reported agreement for fracture classification systems in adults. According to Landis and Koch, a kappa value of 0.66 would be rated as substantial agreement [32]. It is reasonable to believe that the reliability of the fracture classification will improve in the clinical setting, where information about the patient is available.

The number of categories will affect the reliability of any classification. This is obvious if we think of a classification with only one category. Adult fracture classifications have often many categories due to the various fracture patterns that can occur in brittle bone, such as intraarticular affection and comminution. For example, the AO classification of distal radius fractures in adults has 3 types, 9 groups and 27 subgroups, and the reliability has been reported to be less than satisfactory by several authors [6-11] (Table 1). However, intraarticular fractures and severe comminution are rare features of pediatric fractures. It is therefore possible to reduce the number of categories and increase the reliability of the classification without loss of prognostic value. For example, the Gartland classification of supracondylar humerus fractures in children has only three categories, and has one of the highest reported kappa values for interobserver reliability [34].

There are very few fracture classifications for pediatric fractures compared to the vast array of different classifications that exists for fractures in adults. The Arbeitsgemenischaft für Osteosynthesefragen (AO) has recently proposed a comprehensive fracture classification system for pediatric fractures [35]. This fracture classification contains categories for fracture types that are unique for pediatric bone, such as bowing fractures and growth plate injuries. However, this classification does not make the distinction between the buckle (torus) and the greenstick fracture of the distal radius. It is generally agreed that these two common pediatric fracture types are different entities which behave differently and need different treatment and follow up [18,24,25,36,37]. In addition, the AO group has added ligamentous avulsion injuries of the wrist as a separate category. This is a very rare injury in children and was not identified when the AO classification system for pediatric fractures was validated [38].

The AO group reported a kappa value of 0.70 for metaphyseal fractures of the distal radius [38]. However, in this study there were only two categories; complete fractures and buckle/greenstick categorized together. Epiphyseal fractures were analyzed separately. The authors defined the correct classification as that defined by most raters, and then excluded the epiphyseal fractures when analyzing the reliability for metaphyseal fractures. This raises a few concerns: A fracture classification should include all possible fracture categories for that bone (distal radius). When confronted with an injured wrist, the clinician does not know if the physis is involved before the radiological examination. There is often disagreement between raters whether the fracture involves the physis or not. This is certainly the case in our study, as is demonstrated in Figure 2. If we excluded all the growth plate injuries as defined by most raters, there would still be raters that would categorize some of the remaining fractures as physeal. Furthermore, buckle and greenstick fractures should be managed differently [18]. By placing these fractures in the same category the classification will not offer helpful guidelines to the clinician. In addition, it is important that the sample is representative of the study population, since the kappa statistics will vary according to the prevalence of the categories under study [39]. When the number of categories is reduced by excluding one type of fracture, this will change the prevalence of the different fracture types in the sample compared to that of the population at risk, and thus changing the kappa statistics. It is therefore essential that the included fractures come from an unfiltered consecutive series to make sure the sample is representative of the population. This is specifically important when examining the reliability of distal radius fractures in children, since the distribution of categories is highly uneven, with buckle fractures representing the majority of cases.

Our results demonstrate that the fracture classification is not only dependent on the number of categories and the prevalence of the categories in the study population, but also on the experience of the raters. Ideally, a classification system should be simple and independent of the experience of the rater. However, the effect of experience on reliability has previously been described for other classification systems [2,6,40]. The effect of the experience on this particular classification is noteworthy, since these fractures are considered benign and are generally treated by the youngest doctors. The best result at the first reading in our study was achieved by the orthopaedic consultants. It is worth noticing that two experienced consultants had lower intraobserver agreement than the senior registrars. The senior registrars have several years of experience in fracture management, and are involved in fracture classification on a daily basis. At our institution the consultants are in general not involved in fracture management of the distal radius in children, except occasionally while on-call. It seems that both daily fracture management and general experience in orthopedics enhances the reliability.

Stable distal radius fractures in children are extensively monitored with both clinical and radiological follow ups [18,41]. In this series of 105 consecutive fractures, 65 fractures were by consensus defined as buckle fractures. These stable fractures were given a total of 72 clinical follow-up examinations and 34 further radiological examinations. These could have been avoided with more focus on the fracture classification and better supervision. The junior registrars had statistically significant lower kappa value for interobserver reliability than the more experienced raters. They placed fewer fractures in the buckle group, and rated more fractures as greenstick or physeal injuries. This generated more unnecessary follow-ups, but didn't risk any adverse outcome. We coclude that junior registrars overdiagnose, and safe-guard themselves by placing more fractures in categories that merit a follow-up. We encourage the junior registrars to ask for a second opinion. We can avoid an appointment in an overbooked fracture clinic, the child can stay in school and the parents don't have to take time off work to take the child to hospital.

### Limitation of the study

All raters in this study were selected from one institution. This may limit the generalizability of the results to other institutions, thus reducing the external validity of the study. The type and amount of instruction for each rater prior to enrollment in the study is unknown. However, at our institution no systematic instruction for classification are given to doctors treating these fractures, and we have no reason to believe that this is different at other institutions. Only one of the consultants was trained in pediatric orthopedics, and thus the findings may not be relevant for institutions where specialists in pediatric orthopedics are involved in outpatient fracture treatment.

## Conclusions

We conclude that the classification tested in this study is reliable and reproducible when applied by raters experienced in fracture management. More focus on the different fracture categories and better supervision of our younger colleges (who treat most of these fractures in the fracture clinic) will reduce the number of fractures that are considered in need for follow up. This is supported by the results in the study where the more experienced doctor tended to classify better, and where the youngest doctors improved throughout the study period. We recommend this simple four category classification for future research into the treatment and prognosis of distal radius fractures in children.

## Competing interests

The authors declare that they have no competing interests.

## Authors' contributions

PHR conceived the study and participated in the design, collected data, participated in statistical analysis and drafted the manuscript. EAS participated in the design of the study, performed the statistical analysis, and edited manuscript. Both authors read and approved the final manuscript.

## Pre-publication history

The pre-publication history for this paper can be accessed here:

http://www.biomedcentral.com/1471-2474/13/6/prepub
